# Medical Notes and Reflections

**Published:** 1839-10

**Authors:** 


### Art. XIII.

*Medical Notes and Reflections.* By Henry Holland, m.d. f.r.s., &c. Physician Extraordinary to the Queen.—*London*, 1839. 8vo, pp. 628.

This volume consists of a number of detached essays on various subjects relating to the philosophy and practice of medicine. Taken collectively, they belong to a class of writings which are not numerous, because they require for their production a character of mind and a range of acquirement rarely to be met with. Borrowing an illustration from the fine arts, we may compare them to simple designs, which, if drawn by an ordinary hand, would be meager and unimpressive, but which, under the hand of a master, are made to embody just and vivid conceptions, and evince not only more power, but more *learning* than many an elaborate picture on which all the minor resources of art have been expended. The very absence of detail serves to show the truth and vigour of the design: inferior works owe their whole effect to the details. An essay of the kind alluded to results from the mature reflection of a mind naturally capacious, and well disciplined by liberal studies. Such works, in medicine, are nearly peculiar to the literature of our own country. They were more frequent in the last age than they are in the present, and accorded well with a turn of mind conspicuous in the old

English physician—a character whose evanescence from the drama of real life, though perhaps an inevitable consequence of the more general diffusion of knowledge, we, for our own part, shall never cease to regret. In the spirit which pervades them, Dr. Holland's *Notes and Reflections* are redolent of the good old school; they have the same philosophic air, the same tincture of good letters, and the same exemption from the vice of book-making, which constitute leading excellencies in the writings of Mead, and those of Ferriar, more recent in date, but of similar character. While, however, they remind us, in these respects, of some of the productions of the bygone time, they are, nevertheless, perfectly adapted to the present state of science; indeed, it is but justice to our author to say that we have seldom met with a volume treating of so great a variety of subjects, the writer of which has shown himself so completely *au niveau du siècle* in them all.

But we must proceed to give the reader some idea of the contents of Dr. Holland's book. The first chapter, or essay rather, is "on medical evidence."

"There can be few better tests of a sound understanding," says our author, "than the right estimation of medical evidence; so various are the complexities it presents, so numerous the sources of error. The subjects of observation are those in which Matter and Mind are concurrently concerned—matter under the complex and subtle organization, whence vitality and all its functions are derived; mind, in its equally mysterious relations to the organs thus formed—both subject to numerous agencies from without—both undergoing great changes from disease within. Individualities of each have their influence in creating difficulties, and these amongst the most arduous which beset the path of the physician. Few cases occur strictly alike, even when the source of disorder is manifestly the same. Primary causes of disease are often wholly obscured by those of secondary kind. Organs remote from each other by place and function are simultaneously disturbed. Translations of morbid action take place from one part to another. Nervous affections and sympathies often assume every character of real disease. While remedial agents are rendered uncertain in effect by the various forms of each disorder, by the idiosyncracies of the patient, by the difficulty of securing their equal application or transmission into the system, and finally by the unequal quality of the remedies themselves." (p. 1.)

Such being the complexity of the data on which rational belief in medicine is founded, it is not wonderful that the mass of mankind has ever been prone to the most abject credulity, and a prey to every species of imposture; but it does appear somewhat unaccountable that physicians themselves, in their appreciation of the merits of doctrines and the efficacy of remedial means, have shown themselves scarcely less credulous than the multitude.

"Else whence the so frequent description of effects and cures by agents put only once or twice upon trial; and the ready or eager belief given by those who, on other subjects, and even on the closely-related questions of physiology, would instantly feel the insufficient nature of the proof. Conclusions requiring for their authority a long average of cases, carefully selected, and freed from the many chances of error or ambiguity, are often promulgated and received upon grounds barely sufficient to warrant a repetition of the trials which first suggested them." (p. 2.)

Our author anticipates a very improved era of medical enquiry from the introduction of numerical methods, averages, and statistical statements. These, doubtless, are of inestimable value in hands capable of constructing and using them aright; but we apprehend that such capacity can exist only in those whose minds are already imbued with the principles of just reasoning, and informed with the spirit of universal science; while, therefore, we hail the announcement of more correct methods of investigation, we have yet to lament the too limited extension of that frame of mind which alone can render them available, and which is only to be attained by directing the path of the medical student through the fields of general philosophy. *Διο σχεδον των τε περι φυσεως οἱ πλειστοι, και των ιατρων οἱ φιλοσοφωτερως την τεχνην μετιοντες, οἱ μεν τελευτωσιν εις τα περι ιατρικης οἱ δε εκ των περι φυσεως αρχονται περι ιατρικης.** 

Dr. Holland concludes his chapter on medical evidence with a few remarks on undue scepticism. This is a real evil, but of minor extent, as well as less frequent occurrence, than credulity. The same remedy applies to both of these vicious conformations of mind. Habits of cautious induction and disciplined exercise of the reasoning powers will teach us alike to embrace what is true, to reject what is false, and to suspend our judgment on what is doubtful—in which third category a large proportion of what we call "medical science" is still unhappily included.

Chapter ii. is "on hereditary disease." Our author here admits, as the basis of his reasoning, the general law developed by Dr. Prichard, "that all original or connate bodily peculiarities tend to become hereditary; while changes in the organic structure of the individual, from external causes during life, end with him, and have no obvious influence on his progeny." This law, however, is recognized only as a general one, subject to many exceptions, as in those instances where necessities of situation alter certain parts of the organization of animals, and where the continuance of such altered structure is needful for their preservation under new circumstances; a case of which the domestication of animals affords a familiar example. The hereditary tendency to disease shows itself either in the anormal conformation of particular organs and textures, or in the presence of various morbid products; which products may be conceived to owe their origin to the variations of organic texture, or to transmissible peculiarities in the blood and fluids. With respect to hereditary peculiarities of texture, the scope of Dr. Holland's argument is to show their dependence on laws similar to those which give rise to the more marked anomalies of organization or monstrosities. In pursuing this theme he displays an intimate knowledge of the doctrines of transcendental anatomy. This new and extraordinary study has grown gigantic, even in its infancy. It has flung down the barrier between physical and abstract science, by indicating points where they blend, without being lost in each other; and in presenting to the mind a rational type of material forms subjected to our senses, has in some sort realized the subtleties of Plato, and revealed to us the *idea* stamping its impress upon matter. It has already extended its influence into every department of physiology and zoology; and it is difficult to conceive the possible limits of its application. Few attempts, however, have hitherto been made to bring it to bear on pathology, although there can be little doubt that it has, in reality, the most important relations to this branch of science. M. Serres appears to have entertained the notion of such

* Aristot. Lib. de Sensu et Sensibili, cap. i. Ed. Duval.

relation more distinctly than any former writer; but Dr. Holland, in the paper before us, has actually encountered the subject, and much credit, we think, is due to him for the bold yet judicious manner in which he has handled it. The following observations on hereditary tendencies to disease, based on the principles of philosophical anatomy, evince much condensation of thought:

"Examples of the anormal conformation of particular organs, transmitted by descent, are alike numerous and familiar. If peculiarities of external form and feature, whencesoever originally derived, tend so speedily to become hereditary,—affecting, as we see on every side, not families alone, but, by intermixture and descent, whole races of mankind,—we can have no doubt that deviations of internal structure, (whether they be of deficiency or excess, or of any other nature,) are similarly transmitted; and with them propensities to, or conditions of, morbid action in the parts thus organized. Though the direct proof is not equal for the two cases, and though the effects resulting are of such different importance, yet is it certain that the peculiarities, so carried on from one generation to another, have reference for both to one common law. And it is to the same principle that we must look for explanation of the difference in the average duration of life in different families;—a fact well attested in itself, notwithstanding the many exceptions which the laws or social usages of mankind are ever inducing upon it. Those deviations from the primitive or common type of the species, which occur chiefly in the bony structure, integuments, or muscular fabric; producing varieties in the outward form and feature, in the texture or colour of the skin, hair, &c., may exist to great extent, without affecting in any important way the health or natural functions of the individual. On the other hand, much smaller deviations from this type, in the internal organs of circulation, respiration, digestion, absorption, or secretion,—or in the brain and nervous system,—may produce morbid actions, painful in progress and fatal in result: each class of deviations alike transmissible to progeny, under the same general law. This distinction is obviously one of summary importance in the history of disease, and capable of very wide application. It throws light upon the connexion of various morbid states, by giving the relation to a common physical principle in their cause; and this principle one which is associated with other of the more general laws of life. There is no reason to doubt that hereditary peculiarities of structure are as frequent and varied, perhaps as extensive, in the internal organs, as in the external parts of the body. Analogy would suggest this as probable, and it is confirmed in great measure by observation; more extensively as the examination is rendered more minute. On the same grounds it may be presumed that there is a general ratio between the resemblance of external features and that of internal parts of structure. The child most like its parent in traits of countenance and figure has probably closest kindred with him in other and more minute points of conformation. The evidence here is chiefly that of similarity of morbid affections in such cases—a fact almost indisputably ascertained, and which, if the views contained in this chapter be correct, affords the best proof we can reasonably seek for. In the instance of gout it has been observed, that the children most resembling the gouty parent have greatest liability to the disorder. This can only be explained by supposing a corresponding likeness in those parts of internal structure which are chiefly concerned as causes or seats of the disease. It must be admitted, indeed, that this enquiry is still incomplete in many of its parts; and, on first view, it might appear that such internal deviations were much less extensive than those of outward conformation. But we can scarcely name any organ of importance which does not afford evidence of diseased actions, derived from structure and transmissible by descent. And looking to the textures more widely diffused through the body,—as the different vascular systems, the nerves, &c.,—we have every reason to suppose, though the proof be less direct, that they are subject to hereditary variations of structure, not merely in detached parts of each system, but throughout those minute branches and terminations where the most important functions of the body, both animal and vital, may be presumed to take place. On a subject of this nature, however, it is not sufficient to refer merely to the vague division of external and internal parts. The distinction between the animal and vital organs is at the foundation of every such enquiry, and each particular question must be brought into connexion with it. The fact may be considered as ascertained, that the vital organs are subject to more frequent and extensive deviations from the natural type than those of animal life; the principle of symmetry (essential it may perhaps be deemed) in the latter, requiring, for the integrity of the functions, that all such deviations should be limited in extent. It is a remarkable attestation of this fact, that where anormal varieties of the muscles occur (a much rarer event than in the vascular system), there seems a strong tendency in these varieties to become symmetrical for the two sides. A presumption might hence arise, that the tendency to transmission by descent would follow the same law, and the parts belonging to organic life offer more frequent cases of hereditary malconformation than the animal organs. I know not that this question of relative frequency has ever been explicitly answered. There is, however, enough of proof to make it probable that no disproportion exists of the kind just indicated; and that anormal structure in the parts of animal life is quite as liable to be transmitted as in the organic. Taking the obvious instances, indeed, these may appear more numerous; but as the difficulty of observation is much greater in the latter case, it is enough to rest in the fact that both the great divisions of life are liable to this general law, and probably not in any different degree." (pp. 16-19.)

The author adds some curious examples of hereditary defect, which have fallen within his own observation, and suggests the probability of a similar origin of some endemic diseases, as goitre and plica polonica, the prevalence of which, in certain regions, has received no satisfactory explanation from local circumstances or modes of life. He is inclined to refer the great frequency of stone in the bladder, in certain districts, to a like cause, and considers the probability of this view as increased by the well-known connexion of the calculous with the gouty diathesis.

The following is a curious observation, made by Dr. Holland, with respect to *trismus nascentium.*

"When in Iceland, in 1810, I had the opportunity of collecting some facts as to the singular frequency of this disease in the Vestmann Isles, on the southern coast of this island. On these desolate rocks, the population of which does not exceed 160 souls, I found that, in a period of 25 years, 186 infants perished of this disorder, under the age of 21 days; of which 161 died between the fourth and tenth days after birth; 75 on the eighth day. Though the condition of life of these poor people is singularly destitute, fish and the eggs of sea-fowl being their sole aliment, yet is it not so different from that of the Icelanders of the mainland as to explain the frequency of this fatal disorder among them; and it would seem as if some constitutional and hereditary causes were concerned." (p. 22.)

In this instance we think that some local cause, operating on the nervous functions of the individual, is likely to be much more influential than hereditary vice of conformation, especially as such causes may readily be conceived to abound in islands of volcanic origin. In general, however, admitting the premises with which we set out—that all congenital peculiarities of structure tend to become hereditary, and that many morbid dispositions are connected with peculiarities of intimate structure—it becomes evident that hereditary predisposition must have great influence in the perpetuation of endemic diseases, since the extrinsic causes which first impress a peculiarity of structure must operate on each successive generation with the increased force derived from a transmitted susceptibility.

As a general expression of facts, Dr. Holland considers it established "that no organ or texture of the body is exempt from the chance of being the subject of hereditary disease,—or, in other words, every part is susceptible of deviations from the normal type or natural structure, capable of being conveyed to offspring, and of producing morbid actions, which are thus, under the name of disease, propagated through successive generations." (pp. 22-3.)

A singular variety of this law is that which has been called *atavism;* where an anomaly or disease, existing in a family, is lost in one generation, and reappears in the following. Dr. Holland remarks that this, in some cases, may depend on change of sex, and cites an instance known to himself of hydrocele occurring in three out of four successive generations in one family, the omission being in the person of a female, in whose son the disease reappeared. (p. 20.) He well observes that there may be analogous cases, in which the structure affected by hereditary disease is such as to be disguised or superseded by other casualties in the bodily conformation of the individual. There are numerous instances, however, where sex and all other obvious circumstances being the same, the anomaly or disease is missing in one or more individuals of a family series and recurs in their children. It is with the external lineaments as with the peculiarities of internal structure, and we often find some strongly-marked feature of face or form lost in one individual of a family and restored in his offspring. Physiology is entirely at fault in the solution of such phenomena; but, says Dr. Holland, they all concur to establish an unity of plan and a general relation among them, "which makes the simple resemblance of an external feature the exponent of other cases, in which the most severe diseases are conveyed from parent to offspring." (p. 23.)

Another inexplicable circumstance in the transmission of disease is that where several children of a family are affected in common with some malady of which no indication has appeared in either of the parents.

"An example," says our author, "has lately occurred to me in one family, of three sons and a daughter, every one of whom underwent an attack of hemiplegia, before the age of forty-five, though neither father nor mother had been similarly affected. I find another instance in my notes, where three brothers severally suffered hemiplegia, and about the same period of life, without any record of the like event in the family. I have recently seen a fatal case of cerebral disease, with epileptic fits, in a young lady of twenty-four, two sisters of whom had died about the same age, with similar symptoms, though neither parent had been subject to such disorder. I am acquainted with a family in which four children have died during infancy from affections of the brain, without any like instances in the family on either side. In another family, without any similar disease in the parents, three or four children had epileptic fits. I have notes of several similar instances: chiefly, as I think, but not exclusively, disorders of the brain and nervous system. I have known three cases of diabetes mellitus in brothers, under ten years of age, in the same family; one of them fatal in result. In another instance, four cases of ascertained disease of the heart, all fatal about the same period of life, occurred to my notice in the brothers and sisters of one family, without any suspicion, as far as I could learn, of the parents having been the subjects of this disease. By a singular coincidence, another instance is known to me, where four brothers died, between sixty and sixty-five, of ossification and other disease of the heart: but here there appear to have been prior cases of the same kind in the family. In the instance of the deaf and dumb, already referred to, the examples are frequent and curious of several children being thus affected (five out of a family of eight, four from a family of seven), without similar defect existing in the parents. At the school for the deaf and dumb, in Manchester, in 1837, there were forty-eight children taken from seventeen families, the total number of children in these families being 106; and giving, therefore, an average of nearly three such cases in each family. Out of these instances there appears but *one* in which the defect was known to exist in either parent; and we may rightly, therefore, consider this as one of the most striking examples of the fact under consideration." (pp. 24-5.)

The remainder of this essay is occupied with points of great interest, but very difficult solution. Such is the enquiry into those diseases which appear to depend mainly on the formation of morbid products, and the pathology of which involves the question how far the tendency to the formation of such products resides in a peculiar organization of the solid parts, and how far in a taint of the blood. In reference to this question allusion is made to the subject of gout—which is more largely commented on in a subsequent essay—that of scrofula; the pellagra of Lombardy; and other affections. Among the hereditary disorders of the brain and nervous system, insanity holds the most conspicuous place. Our author does not dilate on the subject, but suggests, in a note which we transcribe, a notion highly worthy of attention.

"Here again, as so often before, we are called upon to note the close relation of particular morbid phenomena to more general laws; and, in the present case, to those laws which determine the varieties of character in nations and communities of men. Hereditary deviations in excess, such as come under the character of insanity, are corrected or limited by the usages of society. Those variations from the common type (if we may apply this term to mind as well as body), which are not so controlled, may, in the infancy of any community, and in combination with other causes, become the basis of those more permanent traits which we designate as the character of a people. Such diversities existing, as they actually do, and being in many cases perpetuated within human record, must be derived in some part from this source. Or if we could suppose it otherwise as to origin, it must at least be admitted that this cause is concerned in their perpetuation among races of men." (p. 32.)

Dr. Holland concludes by enquiring to what extent the changes of organization, or other material causes of hereditary disease may continue their progress through successive generations, and what are the limitations to such changes? In attempting the solution of these questions he again happily avails himself of the analogies afforded by philosophical anatomy; and admitting, as most probable, that throughout the varieties and anomalies of organization there are fixed laws, by which the permanence of each species is secured, so likewise, in the less obvious changes of structure or condition on which the hereditary tendencies to disease depend, there is a certain point, up to which the repetition of the modifying circumstances tends to augment and confirm the variation; but beyond which they are restrained by the more general laws of organization, and the original type of the species, which define their extent "differently, perhaps, in different parts of the structure, but still with an eventual and certain limit to all."

We have dwelt at considerable length on this essay, on account both of the importance and novelty of the subject, and the very able manner in which its outline has been traced by Dr. Holland, who remarks, that a work embracing it in its whole extent is yet a desideratum. Such a work would open a very rich and profitable field of research; but he who would explore it must be a man of no ordinary endowments; for he must be at once a physiologist of the first magnitude, and an experienced, observing, and learned physician.

Chap. iii. is on "Bleeding in Affections of the Brain." It appears to us that a very general error has obtained in the reasoning of pathologists with respect to the symptoms of disease of the brain. This, like other organs, has its own individual properties of texture and modes of nutrition, which are liable to various perversions; but, unlike any other organ except the heart, its influence is so diffused over every part of the frame, that it may be said to exercise an universal function. Now, the error we refer to is that of confounding the effects of deranged function of the brain on the entire organism, with the symptoms of some particular lesion of the brain itself; whereas, in reality, such derangement of cerebral function, with all its extensive consequences throughout the system, may arise from the most opposite pathological conditions of the brain,—conditions which agree in nothing except in disturbing the functions of the great centre of animal life, but hence producing analogous effects on various remote and dependent functions. For example, we find a patient insensible and deprived of voluntary motion, with dilated pupils, stertorous breathing, and convulsions; and we say these are the symptoms of compression of the brain. But they are not so; they merely indicate the lesion or suspension of certain functions of the brain, which may arise from various causes, of which compression is one; and the circumstance that compression is, perhaps, the most frequent of these causes, does by no means connect the symptoms alluded to in a more necessary relation to this than to any of the other causes capable of producing similar derangement of the cerebral functions. Viewing the matter in this light, we doubt much if it would be possible to state the characteristic symptoms of any one pathological condition of the brain, except acute inflammation, in which, as in inflammation of other organs, the nature of the morbid action is indicated by the febrile excitement of the system, and its seat by the local pain.

If the foregoing remarks be just, it is not without reason that Dr. Holland asks, "is not depletion by bleeding a practice still too general and indiscriminate in affections of the brain, and especially in the different forms of paralysis?" He adduces various instances which show that this question must be answered in the affirmative, and that various states of diminished cerebral power produce symptoms which might lead to the destructive use of the lancet. Such are the delirium of typhoid fever; the acute and throbbing pain of the head, and general excitement of the system, consequent on profuse hemorrhage; the vertigo and delirium arising from starvation—the phenomena of delirium tremens; the cases of infantile disease in which symptoms of cerebral excitement follow excessive depletion;* and the experiments of Sir A. Cooper, in which it was found that the tying of the two vertebral arteries brought on various species of paralytic as well as spasmodic affection.

* The pathology of the brain in children seems yet more obscure and intricate than in the adult; for not only do the symptoms of cerebral exhaustion simulate those of excitement, but the state of real and active inflammation in very young children is often strangely masked. According to our own observation, profound coma, dilated pupils, and convulsions, sometimes coexist, from the commencement of the attack, with high febrile excitement of the system; and this in cases where, on dissection, the appearances of acute inflammation alone are visible, without any cause of compression.—Rev.

Some judicious remarks follow on several of the symptoms common to different and opposite states of the brain; as coma, vertigo, lightheadedness, headach, the degree of contractility of the pupil, and the susceptibility of the retina.

The following observations on the treatment of palsy are replete with practical wisdom; and the cautions they suggest, though, as the author admits, familiar to many, are not, in general, sufficiently kept in view.

"Even were the tendency to paralytic seizures as generally lessened by bleeding as common practice could imply, it does not thence follow that abstraction of blood from the brain should be needful or desirable in immediate sequel to such attack. In many cases it is undoubtedly otherwise. The paralysis, when depending on apoplexy, with extravasation of blood or serum, or on other cause of continued pressure, may come on by degrees, and admit of relief in its progress by emptying the vessels of the head. But often it occurs as an instant shock to a portion of the brain or spinal marrow, without any proof of extravasation or obvious cause of pressure;—the shock itself being of momentary duration, though it leaves lasting effects on parts of the nervous system thereon depending. In these cases, and they are frequent, the physical causes of the change are little known to us. There are reasons for supposing that the nervous substance itself is often primarily affected. We have certainly no sufficient proof of mere pressure from fulness of vessels being concerned, to warrant large bleeding, especially after the stroke of palsy has actually occurred. The degree of coma attending and following these seizures is not alone sufficient cause for the practice; and will usually subside without it, where the original attack is not such as to endanger life.

"Looking indeed to the magnitude of the event between, common reason would suggest a doubt whether the same treatment can be desirable immediately before, and after a stroke of palsy. I do not mean to give this the weight of an argument. From the nature of the circumstances, it is extremely difficult to bring unequivocal proof on the subject; but there is much cause to believe that the practice of bleeding in the latter case is often injuriously pursued. The risk, I believe, will generally be less from waiting a certain time,—to observe the effect of what has occurred upon the circulation, the breathing, and the sensibility,—than from hastily taking away blood, at the moment of a great shock to the brain, and before we can rightly appreciate its consequences. This effect upon the greater functions of life gives us in fact the best information we can have in guidance of further practice. But this we forfeit in great part, by the disturbance any large depletion makes in the system, and particularly in the organs upon which these functions depend. The practical importance of this consideration may readily be understood.

"Even where evidence is obtained of the fitness of bleeding soon after one paralytic attack, for the prevention of another, the question still remains as to the manner of this,—whether by copious depletion at once, or by smaller bleedings, repeated as observation may suggest. And this question the practitioner, while prepared for boldness in all fit and urgent cases, is bound always to keep before him; seeing especially that any great excess in the remedy may hurry on the very mischief it is sought to prevent. I believe that in most cases the latter method is to be preferred. It accords better with the state of our knowledge of these disorders; involves no irretrievable step; and in its progress affords the information most requisite to decide how far it should be carried into effect. Paralytic cases there presumably are of such nature, that a few ounces of blood taken away at regular intervals will ward off a recurrence of the attack, which any large and sudden depletion would probably hurry on. The proof here can seldom be explicit; but the presumption is one I have often been led to entertain." (pp. 44-6.)

Chapter iv. is on "Sudorific Medicines." There is reason to believe that what is called perspiration involves two distinct processes, one consisting in simple exhalation from the surface, and therefore purely physical in its nature, the other in a vital process of secretion, which is therefore influenced by the various states of the circulation and nervous functions. The latter only is the point of view in which it can be regarded with reference to the operation of medicines. The general tendency of our author's observations is to show that the relief which accompanies natural diaphoresis in many diseases is not necessarily dependent on such evacuation, and that the sweat may, with as much probability, be regarded as merely the sign of an altered state of the system on which the improvement depends. He instances the supposed critical perspirations which occur in fever, and adverts to the familiar fact, that remission or intermission frequently takes place, with accompanying alterations of the state of the skin, but with little or no actual perspiration succeeding the hot stage. Even in the simple paroxysm of ague, which seems to afford the instance most favorable to the idea of a critical diaphoresis, the sweating stage is sometimes entirely absent, and the febrile paroxysm goes off without it. We are convinced from our own experience in this disease, which has not been small, that there is no necessary relation of cause and effect between the three stages which constitute the ordinary paroxysm of ague. We have occasionally seen the cold stage without the hot or the sweating, and have much more frequently observed the gradual subsidence of the hot stage without any marked perspiration. We are persuaded that the notion entertained by Cullen, and others, of a fixed relation between the different stages of ague is entirely erroneous; and we may, indeed, ask, how is it possible that any such relation should exist, unless the cutaneous circulation bore, in every individual, the same relation to the other functions? Of two men in perfect health, one will run a mile without sweating, while another will be drenched with perspiration before he has run a third part of that distance. If, then, the natural movements of the circulation affect the functions of the skin so differently in different individuals, can it be expected that the effects of its morbid excitement will be more uniform?

Our author alludes also to the frequent occurrence of spontaneous perspiration without any relief, and sometimes with aggravation of disorder:

"Proofs of this may be drawn from common experience in continued fevers, pneumonia, acute rheumatism, and various other diseases attended with fever, where profuse or long-continued sweating often occurs, with doubtful benefit or even manifest disadvantage to the patient. The perspirations in hectic fever, though marking the several periods of remission, yet augment the distress of the patient, and increase his weakness. In the fever attending the epidemic influenzas, the more general state of the skin is that of a clammy perspiration, manifestly not producing any remission of the symptoms. Even in the exanthematous fevers, where it might be inferred from the eruptive symptoms that diaphoresis would be more uniformly beneficial, and a legitimate object of treatment, cases constantly happen where natural sweating is attended with no obvious good; and where the attempt to force it out by medicines or other means is distinctly injurious to the patient.

"Many other instances will occur, more or less directly in illustration of the same point. In those complaints usually termed bilious, and where there is accumulation, or disordered change of secretion, in the digestive organs, perspirations break out often and copiously, without any relief to the system. The same happens in various disorders of the alimentary canal; an effect of the intimate sympathy between this great internal membrane and the surface of the body. A tendency to perspiration is the consequence of purging in any excess; as indeed of all causes which, even without fever, tend to debilitate the body. These instances, though less determinate than those of simple and determinate fever, yet illustrate the same general view. If perspiration be regarded as an index of the change of symptoms, rather than as the cause of such change, it may be expected to occur in many cases, where it does not indicate any favorable alteration, and where in fact none such takes place." (pp. 54-5.)

Dr. Holland remarks that there must be at least two, and possibly more conditions, under which perspiration takes place: the one consisting in simple relaxation of the cutaneous vessels, the other in augmented activity of the same vessels, whereby the fluid is forcibly thrown out.* The former, being the reverse of that which obtains during the hot stage of fever, indicates an entire remission of the febrile state, while the latter is attended with little diminution of it.

He admits, also, that consideration is due both to the quantity and quality of the perspired matter, as ridding the blood of superfluous or noxious ingredients, a view which derives increased importance from those researches which show that a function analogous to respiration is carried on by the skin, and that the quantity of carbon eliminated from the blood may be considerably increased through this channel. The presumed influence of perspiration in reducing animal temperature must also be taken into account; but, as our author observes, the frequent continuance of morbid heat during excessive perspiration proves that there is no relation between the two symptoms which can be of any great value in practice. On the whole, Dr. Holland regards the use of diaphoretics as less determined by method or reasonable experience than that of other evacuant remedies, and lays down the following positions as established by sufficient evidence:

"First, that it is more reasonable, as well as beneficial in practice, to have regard to the changes in the circulation producing diaphoresis than to the action of sweating itself. And, secondly, that the amount of perspiration is rarely a just measure of the good obtained; and that to make this a primary object is likely to give a wrong and injurious bias to the treatment of disease." (p. 62.)

Chapter v. is on the "Effects of Mental Attention on Bodily Organs." This is an ingenious essay on a very curious subject. Dr. Holland commences by observing, that while the influence of the will on the voluntary muscles, and of the passions of the mind on other parts of the economy, have long been studied, little notice has been taken of the effects of consciousness directed by a voluntary effort to particular parts of the body. This concentration of the attention on any one part may, he says, be exercised as a mental act, without the suggestion of previous sensation.

"Though it may be termed a function of the will directed towards the body, it produces no effect on the muscular structure as such. Where indeed the attention is excited by external impressions, it is perhaps but another name for sensation itself; but we need a different term for that voluntary act by which the consciousness receives, as it were, a local direction, and is by effort retained for a time in this state.

* Presuming that the greater part of diaphoretic medicines act by relaxing the cutaneous vessels, we have a good instance of the distinct nature of the two modes of perspiration in the horse. In this animal perspiration is easily excited by exercise; that is, by increasing the force of the circulation, but no medicine will produce a diaphoretic effect. We have frequently met with human subjects who perspired very readily by exercise or heat, but on whom sudorific medicines had little effect.—Rev.

It is this enquiry which I do not find to have been explicitly made; though the familiarity of the effects, and of the language applied to them,—as well as the speculations regarding a vital principle, common to physiologists of every age,—may be said to have implied in reality all the points in question." (p. 64.)

The subject has not, indeed, been sufficiently investigated, but the general truth was distinctly present to the mind of John Hunter, who made also an application of it to animal magnetism similar to that which we shall presently find noticed by Dr. Holland.

In Mr. Parkinson's Hunterian Reminiscences we find the following words of this great physiologist, who had consented to subject himself to animal magnetism:

"I feared lest my anxiety for the event should bring on my spasm, and that should be imputed to animal magnetism; but considering that if any person was affected by it, it must be by the imagination being worked up by attention to the part expected to be affected, and thinking I could counteract this, I went: and, accordingly, when I arrived at the place, I was convinced, by the apparatus, that everything was calculated to affect the imagination. When the magnetiser began his operations and informed me that I should feel it first at the roots of the nails of that hand nearest the apparatus, I fixed my attention on my great toe, where I was wishing to have a fit of the gout; and I am confident that *I can fix my attention to any part, until I feel a sensation in that part.* Whenever I found myself attending to his tricks, I fell to work with my great toe, working it about, &c., by which means I prevented its having any effect on me." (*Hunter.*)

Although the materials for observation on such points are very abundant, inasmuch as every man carries about a store of them in his own person, there are two causes which render it extremely difficult to arrive at any certain conclusions with respect to them: the one is the impossibility of fixing on any definite nomenclature of sensations; the other is the different relations which subsist in different individuals between the nervous system at large and the functions of particular organs. Still we believe that the principle which Dr. Holland seeks to establish has a firm foundation in truth; namely, that continued attention, or in more precise language, the continued direction of consciousness, to particular parts, has a power not only of altering their sensations, but of affecting their functions in a greater or less degree. He adduces a variety of examples in which sensations or actions may be excited by the simple direction of attention to the organs. Thus yawning, coughing, and sneezing, are occasionally excited in this manner. The organs of articulation and deglutition are variously subject to the same influence: a person who stammers will stammer more than usual if he wishes particularly to speak plain, and the act of swallowing is always rendered difficult by fixing the attention on it. A similar direction of consciousness to the region of the stomach causes a feeling of weight, oppression, or other uneasiness; and, when the stomach is full, seems greatly to interfere with digestion. The action of the lower bowels is often excited by attention to them, and a desire to empty the bladder is readily occasioned in the same manner. The last-mentioned phenomenon is very common in highly nervous persons, who, if they see the prospect of being confined for some length of time without an opportunity of retiring, will be seized all of a sudden with a strong inclination to empty the bladder; and we may notice, as a curious fact, that the kidneys often sympathize with the irritability of the bladder thus artificially induced, and secrete a large quantity of urine in a very short time.

"The power which this stimulated attention to particular parts of the body has in altering, not only the sensations derived from them, but also more or less their functional state, may be instanced in many other ways. The salivary glands, for example, are manifestly thus altered in their secretions; organs, it may be remarked, singularly susceptible of being affected instantly by all mental emotions. In the gums, the sensation created by attention given to them may arise almost into pain. The feelings produced in the tongue in like way are peculiar and well marked. Or a single limb, or portion even of a limb, may be taken for experiment; and a peculiar sense of weight and restlessness, approaching even to cramp, be produced by urging the attention expressly upon it. Here the muscular texture may be presumed to be chiefly affected; and with feelings much akin to those generally arising from fatigue, stagnant circulation, or other causes. Sensations of heat or cold, or other more vague feelings, on the surface of the body, may readily be created in similar way. In that state of skin, however produced, of which general itching is the symptom, the attention directed upon any particular part, will very often bring this sensation immediately to it. Such cases as these, where it is difficult to prove more than a change or increase of sensation from the parts under this influence, may appear ambiguous in proof. But that some real alteration is made in them, as respects either their nervous state, or circulation, or both, is probable from the more distinct evidence of this in other instances, from the same cause of excitement." (pp. 67-8.)

"All parts of this subject are curious, and deserving especial notice in their relation to the symptoms of disease. The case of the dyspeptic I have already mentioned. Closely akin to this is the disorder of the hypochondriac; some of the most singular perversions of which admit of the same explanation. Here the patient, in fixing his consciousness with morbid intentness on certain organs, creates not merely disordered sensations, but often also disordered actions in them. There may be palpitation of the heart, hurried or choked respiration, flatulence and other distress of stomach, irritation of the bladder; all arising from this morbid direction of attention to the organs in question. It is certain that many of the secretions are immediately affected by emotions of mind; and the case appears to be the same from anxious and sustained attention to the parts concerned in these functions.

"In chorea, hysteria, and other diseases where disordered nervous actions occur, the same principle is more or less concerned; but without equally distinct intervention of consciousness and the will, as in most of the instances already cited. Yet in hysteria, the instances are frequent of attacks brought on by the mere expectation of them; or by imitation; or *occasionally even by a sort of morbid solicitation of the organs to these singular actions.* Of the latter fact medical experience furnishes extraordinary examples. In diseases of this class, such results are connected with those more commonly recognized as produced by disorder of the sensorium upon the animal and vital organs." (pp. 69-70.)

The author does not hesitate to ascribe some of the results of animal magnetism to the effects induced, in different parts, by concentration of the attention upon them; and he remarks, that when the head or præcordia are the parts so influenced, especially in hysterical subjects, very singular, and apparently mysterious phenomena may result. The fact expressed by the words which we have placed in italics in the foregoing extract, is regarded by Dr. Holland as affording an explanation of some of the more singular incidents of the alleged magnetic state. To this we entirely assent. In a large proportion of the cases we have witnessed, the patients were manifestly impostors; in others, however, the phenomena appeared to be those of real hysteria, determined as to their particular character and locality by an eager expectation and desire of their occurrence. It may be added that, when they have once taken place, habit must exert a powerful influence in their reproduction.

How it comes to pass that the direction of consciousness to a part can thus create sensations and modify organic functions, is one of the most difficult problems in physiology. As Dr. Holland observes, there are several reasons which render it nearly certain that some nervous function is concerned in the production of these results. This being conceded, the question arises, through what class of nerves are such actions carried on? Those of voluntary motion (says our author,) can scarcely be admitted, because motion is no part of the effect, and the influence extends to parts over which we have little or no voluntary power: on the other hand, if we resort to the nerves of sensation, we must recognize two modes of action in opposite directions along the course of the same nerve, but of this we have no proof.

We agree with our author, that we possess no means of solving the difficulty; but, if we might venture on an hypothesis, we should suggest as possible that a *sensation* of the kind alluded to, though referred to a particular part, does not originate in that part, but is, in reality, an *internal sensation* arising exclusively in the sensorium from a repetition of that action whereby former sensations produced by external causes have been perceived. Of the reality of such a case we have ample proof in the sensations which are referred to the extremity of a limb months after it has been amputated, and frequently with the most minute reference to certain parts of it, as a finger or a toe. With respect to the *changes of function* under consideration, we should be inclined, on this hypothesis, to refer them to the agency of the organic nerves influenced by the long continuance of the cerebral actions above mentioned; nor do we see anything unlikely in this supposition, because there is little doubt that the ganglionic system, though its functions be distinct and peculiar, is very much under the influence of the brain. We have noticed this chapter at considerable length, because the subject of it has relation to many obscure points in the pathology of nervous diseases, and has moreover a direct bearing upon some remarkable phenomena which have lately attracted much attention, and given rise to no small exhibition of folly and credulity. These phenomena, now heterogeneously associated under the name of animal magnetism, will probably lead, in the hands of sober enquirers, to the discovery of a new and important tract of physiology. Pseudo-sciences, though they have no truth *in* them, have generally some truth *under* them.

Chapter vi. is on "Points where a Patient may judge for himself." It contains some sound precepts as to the licence to be conceded to patients with respect to their own choice of temperature, diet, posture, and confinement to bed or otherwise, the fitness of particular remedies, and the propriety of protracting or discontinuing the use of medicines. On all these points Dr. Holland believes that the practitioner may often derive knowledge from the expression of the patient's natural feelings and instincts, and concludes that "a discreet forbearance is as needful to the physician as firmness, and the best rule is not to be implicitly subservient to rule, a maxim essential in all cases to the right practice of medicine, seeing the many contingencies which are ever arising in contradiction to its soundest methods and precepts." We strongly recommend this chapter to the attention of routine practitioners, convinced as we are that the adoption of the advice it affords would prevent what very frequently happens, namely, that the patient, in addition to the perils of the disease, runs some risk of being bored to death by the doctor.

In chapter viii. on the " Abuse of Purgative Medicines," will be found some just animadversions on the too prevalent practice of giving constant purgatives, and insisting on daily evacuations, without reference to the natural constitution of the individual. The author remarks that a certain degree of distension of the large intestine is essential to its healthy state, and that the continual use of cathartics, by preventing this, and substituting a partial and unequal distension by air, produces irritation, and impedes the natural peristaltic actions.

He particularly reprobates the habitual employment of this class of medicines in cases of torpor of the bowels arising from debility.

"The colon, perchance, cannot readily or quickly propel its contents, though the earlier stages of digestion are well and easily performed. To remedy this defect, it is goaded by the constant use of cathartics, which injuriously fret the stomach and long tract of bowels through which they have to pass before reaching this part. The habitual irritation of the mucous membrane alters and depraves its secretions throughout the whole course of the alimentary canal, becoming thereby a further source of mischief and suffering to the patient. These disordered secretions are too often urged in proof of the need of further evacuation, (an error sometimes arising from inexperience, sometimes from a graver source;) and thus the practice proceeds, in a vicious circle of habit, from which the patient is rarely extricated without more or less of injury to his future health." (pp. 99-100.)

Dr. Holland observes, that if we seek in such cases to obviate the evil through means which act only by irritation, we shall increase it: the proper plan is to combine tonics with laxatives. There are cases, he says, in which, if there be no irritation (inflammation?) of the mucous membrane, bark will often actas a laxative. The observation is perfectly correct. The author has several good remarks on the use as well as abuse of purgatives. Where this class of remedies is indicated, he advocates the exhibition of full doses, with proper intervals between, in preference to the more frequent repetition of smaller doses, excepting those cases where the vital power is too feeble to encounter any sudden change without risk. We believe the precept to be practically good. The following observation on the treatment of obstruction of the bowels is most just and well worthy of attention:

"I do not mean that purgatives should not be given in cases of obstructed bowels; but I wish to convey the caution, required, as I think, by the too uniform direction of practice in these instances; viz. that if there be distinct local pain, threatening inflammation in any part of the canal, or much active irritation, with nausea and vomiting, and if the first cathartic medicines, freely given, fail of success,—it behoves the physician well to consider whether he shall urge this treatment further. There are doubtless cases where it is expedient to do so; but many others where the irritation of drastic purgatives hurries on the patient to danger or fatal result; and this is not unfrequently, where quiet; abstinence equally from food and medicine; leeches and fomentations over tender parts of the abdomen; or still better, in some cases, leeches to the hæmorrhoidal vessels, would have removed obstructions, and relieved the complaint." (pp. 104-5.)

We are quite convinced that many cases of fatal enteritis originate entirely in a practice opposite to that here recommended.

Dr. Holland observes, with truth, that the abstraction of blood from the hemorrhoidal veins is too much neglected, especially in this country. It is, in fact, the only form of general bloodletting in which a sufficient reason can be given for drawing blood from one vessel rather than another, inasmuch as the portal system is in some degree insulated from the general circulation, and is more immediately concerned in the functions of the chylopoietic viscera. The immense relief which every practitioner must have witnessed from a free hemorrhoidal discharge in various diseases of the abdomen, clearly indicates one of those instances, unhappily too few, in which a therapeutical intention may be directly founded on a natural remedial process. We think, however, that the study of natural therapeutics, as it may be called, is not sufficiently cultivated, and that careful observation and experiment might multiply such instances to a greater extent than is generally believed.

Chapter ix. is on "Methods of Prescription." It contains some judicious remarks of a general nature: these, however, we pass over, and proceed to notice the important subjects of "Gout and the use of Colchicum," with which chapter x. is occupied.

The author assumes, in limine, the following postulates as either ascertained or strongly to be presumed:

"1. That there is some part of bodily organization disposing to gout, because it is an hereditary disorder. 2. That there is a *materies morbi*, whatever its nature, capable of accumulation in the system,—of change of place within the body,—and of removal from it. 3. That though identity be not hitherto proved, there is a presumable relation between the lithic acid, or its compounds, and the matter of gout; and a connexion through this with other forms of the calculous diathesis. 4. That the accumulation of this matter of the disease may be presumed to be in the blood; and its retrocession or change of place, when occurring, to be effected through the same medium. 5. That an attack of gout, so called, consists in, or tends to produce, the removal of this matter from the circulation; either by deposits in the parts affected; by the excretions; or in some other less obvious way, through the train of actions forming the paroxysm of the disorder. 6. That there is intimate relation between the condition of gouty habit, and the functions of the kidneys and liver, both in health and disease. 7. And that the same state of habit, or predisposition, which in some persons produces the outward attack of gout, does in others, and particularly in females, testify itself solely by disorders of internal parts, and especially of the digestive organs." (pp. 116-7.)

The second of these principles, regarding a material cause of gout circulating in the system, and eliminated from it by the gouty fit or in other ways, has been disputed by high authorities; but, as it is acquiesced in by many, is supported by all the facts and analogies furnished by recent enquiry, and affords a readier solution of the difficulties of the subject than any other that has been proposed, Dr. Holland makes it the basis of his reasoning.

Under this view, less importance is attached than formerly to the actual paroxysm of gout, since it comes to be regarded "as one only of a series of changes taking place within the system; though, perhaps, the most characteristic and interesting in its obvious effect of relieving the constitution for the time from the causes of the malady." Admitting a morbific matter as the efficient cause of gout, the question arises whether the hereditary tendency to this disease consist in a disposition to form or accumulate such matter by secretion or retention within the system, or in some peculiarity of texture in the solid parts, rendering them liable to inflammation of a specific kind, and occasioning the deposition of the peculiar matter when abounding in the system from other causes? The former of these opinions is espoused by Dr. Holland, who affirms that we have no reason to regard hereditary gout as more than "a disposition to generate a certain morbid matter within the body, in effect of certain circumstances of structure either favouring its formation or preventing that excretion of it from the system which is essential to a healthy state; or, in other words, gout as an hereditary disease may depend upon some transmitted peculiarities either in the organs of assimilation, or in those by which certain parts are separated from the mass of the blood."

We cannot say that we are inclined to so exclusively humoral a pathology of gout as our author, because, admitting as we fully do the presence of a morbific matter in the blood, the relations between this fluid and the textures which it supplies with the materials of life are so intimate, that we cannot conceive a morbid peculiarity of the blood to be transmitted from generation to generation, without inducing, as a necessary consequence, peculiarities of minute organization in all the textures. Yet, on the other hand, it is perfectly conceivable that other circumstances may so influence the organization of the solids as to render them more or less susceptible of influence from the morbific cause resident in the blood. We think, therefore, that a combination of the suppositions stated by Dr. Holland affords a better solution of the phenomena of gout than either of them taken separately, and especially of those relating to the exemption of certain individuals in gouty families.

Dr. Holland believes that the peculiarities which constitute the gouty diathesis may be generated in the individual independently of any hereditary taint. We would extend this admission to all diseases communicable from one individual to another, whether by consanguinity, contagion, or any other means. Those who deny this seem to forget that every disease must have originated in the person of some one or other, or it could not now exist; and if so, we must either admit that the disease may have a new origin in any other individual similarly circumstanced with the first, or that the first was placed in physical circumstances in which no human being could ever be placed again, a highly improbable, not to say absurd supposition. With respect to the actual nature of the morbid matter of gout, Dr. Holland thinks

"It is probable that the discovery, if made, will show it to be,—not a matter alien to the system, and wholly morbid in kind,—but rather the excess, either from superabundant formation or undue retention in the blood, of some material, a certain amount of which is incompatible with, or even necessary to, the health of the body. Or this view may be modified in part, by supposing that, though generated in the body, it is so, only as an excretion needful to be removed, and hurtful in its retention or accumulation there. I have already alluded to what at present is the most plausible conjecture on the subject. Without venturing to antedate our future knowledge, by expressly defining the matter of gout to be either lithic acid, or urea, or one of the lithic or purpuric salts, or any other highly azotised principle, it is impossible not to suppose that there is produced in the blood some animal principle having close kindred with these, and morbid either in kind or by excess;—a matter in the separation of which the kidneys are largely concerned, and the retention of which in the system is the cause of various disorders, according to the age, sex, temperament, or other peculiarities of the persons affected." (pp. 128-9.)

He is of opinion that researches pursued in this direction are likely to lead to a more intimate knowledge of the disease in its active form, and also of its connexion with other local or constitutional disorders.

"Modern observation has led us to recognize some of these relations under the names of gouty headach, gouty ophthalmia, and gouty bronchitis. My own experience would lead me to add certain forms of asthma to the number. But many more undoubtedly yet remain to be determined; and not the least important, those which subsist between gout and the system of the brain and nerves. Reference has already been made to hypochondriasis; and it is highly probable that other disorders of the same class, still less generally viewed under this connexion, will hereafter be submitted to it.

"The relation of gout to the functions and disorders of the liver, is another point of much interest in pathology,—clearly attested both in the active symptoms of the disease, and by those which are common under other forms of the gouty temperament. Its connexion with cutaneous diseases is an additional topic, yet almost unexamined; though I cannot doubt, from my own observation, that certain of these disorders occur as effects of the habit in question. I have so often seen psoriasis, for example, prevailing in gouty families,—sometimes alternating with acute attacks of the disease, sometimes suspended by them, sometimes seeming to prevent them in individuals thus disposed,—that it is difficult not to assign the same morbid cause to these results, however unintelligible its mode of action under such different forms.

"But the kidneys, as already stated, are evidently the organs of the body, upon the disordered or deficient action of which depend those changes in the circulating fluids, which have closest relation to all the phenomena of gout. These functions, it is important to observe, undergo variation at successive periods of life, independently of actual disease. By such variation they serve in part to the destined changes of the body at these respective periods; this influence being attested by an altered state of the secreted fluid, both in the nature and proportion of several of its ingredients. That period which begins the decline from perfect manhood is marked generally by an excess, if it may be so termed, of the lithic acid, which continues more or less through after life;—testifying itself with the greatest safety, and often remedially, by large habitual discharges of this substance from the kidneys; becoming a source of grave and various disease where this separation is insufficient or suddenly interrupted. Much certain discovery for the future (perhaps even as respects the causes and phenomena of fever) may be affirmed to lie in this particular path of physiology. And much more of practical caution might be drawn, even from our present knowledge, as to interference with these important functions, whether in health or in the treatment of disease." (pp. 129-130.)

Some other points of considerable interest are adverted to by Dr. Holland, which lack of space prevents our noticing. He subjoins to this essay some remarks on the use of colchicum. And first he enquires, does its operation consist in destroying the matter of gout by some specific change, or in withdrawing it from the part affected into the general circulation, or in removing it from the system through some of the excretory organs? These, he observes, seem to include all the modes in which it can be supposed to act, except the improbable one of its exclusive influence on the nervous system. Experience affords ample proof of the efficacy of colchicum in every modification of the disease, in whatsoever texture it may be situated; and we are hence naturally led to infer its action on the morbific matter diffused throughout the system: but this inference by no means implies any chemical or other change immediately effected on the matter itself; it is more probable that the medicine exerts its specific power on some organ the function of which is expressly connected with the morbid conditions of gout. He thinks that colchicum acts on the kidneys more decidedly than on any other part, and this independently of the presence of any gouty disease or disposition. On the nature of the changes induced by it in the urinary secretion he has not been able to satisfy himself, though he is convinced that its operation is not confined to a mere increase of quantity, but involves a change in the nature or proportion of the animal compounds excreted. This idea accords well with the pathological views already stated, with the presumed nature of the morbific matter, and with the acknowledged affinity of the gouty and calculous diatheses.

Such a view, if admitted, affords some important practical suggestions as to the administration of colchicum, and especially as to its use as a preventive. Dr. Holland has no belief in the opinion that this medicine renders the recurrence of the paroxysm more frequent. This opinion he believes to have arisen from mistaking the effects of the disease for those of the remedy, and from the faulty or insufficient application of the latter; and he attributes such misapplication to the too exclusive attention given to the external development of the disease. The medicine has been used empirically to remove the local inflammation, and discontinued when this was effected; whereas it admits of continued use on the definite principle of eliminating the morbific matter from the system: to attain which end it should be conjoined with means adapted to sustain all the excretions in a sufficient degree; this being the best guarantee that the matter is fairly carried out of the circulation.

We regret that we cannot follow out our author's observations on this subject; but we have stated above the principle which pervades them: we think that it well deserves to be kept in view, and made an object of enquiry both by the animal chemist and the physician.

Chap. xi. is on "some supposed Diseases of the Spine." Some good remarks will here be found on a class of cases in which disordered function of the spinal nerves is liable to be mistaken for organic disease of the spine. These cases are most frequent in females, and especially those of an hysterical habit. There is no doubt that a misunderstanding of their true nature formerly led, and does frequently still lead to much injurious practice in the way of confinement to the recumbent posture, local depletion, and severe counter-irritation, all of which tend, in the most direct manner, to aggravate the disorder. The author does not lay claim to any originality in his view of the subject, and we shall not enter into it further than to express our satisfaction at one of the curative measures he enjoins, namely, "exercise of the limbs sedulously persevered in and extended." We believe that a frequent effect of that bodily indolence which is one of the greatest evils of civilization, and which obtains particularly in the female sex, is to exalt the functions of the sensatory nerves at the expense of the motor tracts of the spinal cord, the functions of the latter being enfeebled by not being called sufficiently into action. At all events we can speak from manifold experience to the practical truth that properly regulated muscular exercise is the most powerful remedy in some cases of spinal irritation.

There are several chapters in this volume which treat respectively "of the Brain as a Double Organ," "on Dreaming, Insanity, Intoxication,"

on "Sleep," on "Time as an Element in Mental Functions," on "Phrenology," and on the "Present State of Enquiry into the Nervous System." These essays are so connected in their subjects that it would be difficult to insulate one or more of them for particular examination. We omit all detailed notice of them at present: first, because the purely physiological part of them will come under review on an early occasion along with the works of some other authors; secondly, because many of the topics they involve belong as much to psychology as to medicine; and the practical character of this Journal obliges us to exclude such subjects, except on particular occasions. We would not, however, for a moment be thought to depreciate abstract science as a study for the physician: on the contrary, we sincerely regret that the greater number of medical writers evince so little familiarity with it, and there is nothing which we admire more, in these essays of Dr. Holland's, than the evidences they afford of an assiduous addiction to mental philosophy.

Chap. xiii. is on "some Points in the Pathology of the Colon." Our author here offers the following suggestions: First, that the large intestine is more an organ of secretion than of absorption, and that its secretions are not merely subservient to changes in the matter passing through it, but useful or necessary in removing other excrementitious matters from the system: whence some important considerations as to the treatment of certain forms of diarrhœa, such as whether the diarrhœa should be checked or allowed to proceed, and whether practice should be directed to the gradual alteration of the secretions or the immediate removal of noxious matters. Secondly, that many morbid states of the alimentary canal which are referred to the stomach or liver have their real seat in the colon—a fact little noticed in books, but of which we conceive no practitioner endowed with any observation can have the smallest doubt. Thirdly, that, owing to the attachments of this portion of intestine and its immediate proximity to several important organs, its unequal distension and frequent changes of position give rise to various sympathetic affections, the nature of which is frequently misunderstood. The most important remarks are those on the morbid secretions of the large intestine. This subject, however, has been too little investigated to afford data for any critical examination of Dr. Holland's views, which, indeed, are advanced only in a very general manner. On the whole, we regard this chapter as well worthy of attention, and indicative of several points on which there is need of enquiry.

Chapters vii. and xiv. treat on the "Connexion of certain Diseases," and on the "Epidemic Influenzas of late years." We notice these chapters together because they have a common tendency.

Dr. Holland's observation has led him to trace a singular concurrence of measles, scarlatina, hooping-cough, and infantile remittent fever, in particular districts about the same period of time; and he has noted also, as a variation of this fact, that one of these disorders is often exceedingly prevalent in certain localities, while another is equally so at the same time in contiguous places. He alludes to the familiar fact that scarlatina and measles have an affinity which sometimes renders them difficult of discrimination, and further remarks that these diseases have been very frequent during the course of influenzas, at which times a class of cases of a singular and ambiguous character present themselves, having many appearances analogous to each of the exanthematous disorders above mentioned, but chiefly, as he thinks, to scarlatina. He notices the tendency of erysipelas and puerperal fever to assume an epidemic form in the same seasons, and the increased frequency of both these disorders during the prevalence of influenza. He observes that hooping-cough and infantile remittent fever are both remarkably coincident with influenza; that hooping-cough rarely prevails as an epidemic without a simultaneous prevalence of those bowel affections attended with fever, which, in their more distinct forms obtain the name of infantile fever; and that ulcerations or eruptions of the mouth, fauces, nose, lips, and face, are very frequent, especially among children at those periods when disorders of the bowels, whatever be their cause, are particularly prevalent and severe. The dysentery of adults, he continues, has various relations to infantile fever in the course of the symptoms, in the textures affected, and in the lesions consequent upon it; and perhaps there may be no differences between the diseases beyond what are compatible with a common cause acting under different conditions. He extends this remark to the suggestion that the disorders of childhood already mentioned may have a virtual identity with certain fevers or other diseases of adult age, the operation of the causes varying under altered circumstances. Is this view quite in unison with an opinion expressed in chapter xxiv. on "Diseases commonly occurring but once in Life?" It is there maintained as probable that the series of actions or changes constituting such diseases have their seat in the blood, and are carried on throughout by the circulation, and that the future insusceptibility arises from a change in the state of the blood. The first position—that the essence of these diseases consists in a virus acting on the blood—can hardly be called in question; but if the diseases reappear with a new face in the adult, it follows that the blood must, in some sort, retain its susceptibility of the action of the virus; and we should be rather led to ascribe the difference of its effects to an altered constitution of the solids. It is scarcely fair, however, to argue on what is thrown out only in the form of suggestion, or to insist on too exact a congruity between ideas which, in the present state of our knowledge, can at best be but dimly defined. Dr. Holland observes that dysenteric symptoms are generally prevalent along with the diseases of childhood just alluded to. He has also found that dysentery and other bowel affections become much more frequent on the decline of catarrhal epidemies. It is doubtful whether this should be regarded as a translation of morbid action from the same original cause, or merely as an incidental effect to which the system becomes liable from the previous disorder: Dr. Holland inclines to the former supposition. He finally comments on the analogy which influenza bears to the milder forms of typhus and to intermittent fever. The intermittent character of the epidemic catarrhal fever is strongly insisted on, and is supported by the testimony of Sir George Baker, who describes the same type of fever accompanying the influenza of 1762. It will easily be perceived that Dr. Holland's aim is to establish some real relation, however indefinite, among a number of different diseases, and to bring them all into apposition with influenza, in the hope of deriving from the contemplation of the last-mentioned epidemy some principles of general application and utility.

"In referring thus frequently to these epidemic influenzas, which have so frequently and widely prevailed of late years, I may add, that no class of diseases lays open to us a larger field of practical enquiry, as relates not only to their own nature, but also to the connexion of other diseases, with which they are closely associated. The simultaneous or rapidly successive influence of a common morbid cause over large communities and countries discloses relations which in no other way are equally accessible to research. In showing the various forms which a single disease is capable of assuming, it illustrates the nature and action of the circumstances which thus modify it, and especially the effect of particular textures in altering the aspect of the symptoms. We have not sufficiently drawn from this source of knowledge. It is probable that we may hereafter learn from it the virtual identity of many diseases; hitherto placed asunder by distinctions which have foundation only in subordinate symptoms, thereby disguising from us what is most important both in pathology and practice. Or, if no such identity be proved, we may find evidence, scarcely less curious, of an endemic state of constitution (be it called adynamic, or by any other name,) which, originating with the same causes that produce the symptoms of influenza, renders the body for a period more prone than usual to certain other disorders, the material causes of which are ever more or less present. Each mode of viewing the subject is probably correct in part, and they are perfectly compatible with one another." (pp. 92-3.)

The idea is good, and far be it from us to damp the ardour of enquiry; but the perusal of the chapter on influenza is sufficient to show that the benefits to special pathology from the study of this widely-spread disease are yet in reversion; for our author's remarks as to its origin and nature consist chiefly in a statement of difficulties, conjoined, however, with a judicious exposition of the course of enquiry most likely to lead to their solution. His precepts as to the treatment are, we think, in general sanctioned by the experience of the best practitioners. One of them, relating to the use of the sulphate of quina, is founded on the prevalence of an intermittent type in this disease which appears to have presented itself more distinctly to Dr. Holland than to many other observers, and more so, we confess, than to ourselves.

"Any inference that might be drawn, as to its use, from the tendency in the disorder to these intermittent actions, is fully justified by the actual effects. It relieves them, when fully established, almost as speedily and certainly as the attacks of a common ague; and this whatever the part of the body so affected. This remarkable power over one of the conditions of the disease gives so far a specific character to the remedy, that it may rightly be adopted in prevention of a state which it is capable of curing. It is not easy to define an exact time at which its use should be begun. This must vary in different temperaments, and in different degrees of the disorders. But it is an inference from the reasons already stated, that quinine may be given safely and beneficially in many cases where there is still hard cough, with pain, oppression, and restlessness;—and experience confirms this conclusion. A soft feeble pulse, and moist skin, often concur with these symptoms, and furnish additional authority for the practice. If the cough itself, as frequently happens, tends to intermittent character, the security of the remedy becomes greater, and its effects more speedy." (p. 211.)

Chapter xviii. contains some brief but just comments on the "Method of Enquiry as to Contagion." The author refers, for a full exposition of the laws of contagion, to a paper by the late Dr. Henry in the Fourth Report of the British Association. A review of it will be found in the Second Number of this Journal.

Chapter xix. is on the "Medical Treatment of Old Age." The subject is introduced with some appropriate physiological remarks, in which, however, the author leans more than we are disposed to do towards the belief that merely physical laws enter largely into the arcana of life. We think the phenomena of old age afford the strongest case in favour of an opposite opinion. As age advances physical laws assert a gradually increasing power in the organism: why? Because the vital energy by which they are controlled is becoming progressively more feeble. Death resigns all to the domain of physics.

Some of the best practical observations in this chapter have reference to those habitually increased excretions, common in old age, which our author regards as depurating rather than morbid processes; and hence inculcates caution in interfering with them. He instances particularly the augmented discharge of mucus in the bronchial affections of old age, and the increased excretion of lithic acid with the urine. He notices, also, as of less frequent occurrence, but still worthy to be kept in mind, under the same point of view, the passive hemorrhages incidental to old age: epistaxis, hematuria, hemorrhages from the bowels, uterus, &c.

The consideration of these subjects carries us back into the Greek era of medicine, which we are convinced is destined to diffuse much light over the future annals of the science.

There is a remark concerning the pulse, which we notice because the fact to which it refers, though very obvious, is too often overlooked in practice. We allude to the jarring stroke of the pulse caused by organic affections of the heart, so common in old age, and which is liable to be mistaken for an indication of strength. This applies particularly to the treatment of apoplexy—a disorder of advanced life, and one which is very frequently indeed associated with disease of the heart. It has occurred to us more than once to see a practitioner fully prepared to take away thirty or forty ounces of blood, in a case of apoplexy, but astonished to find the pulse sink to a thread before ten ounces had been withdrawn. Dr. Holland expresses his belief, in a note, that there is no important structural disease of the heart which is not represented in some manner by the pulse, as felt in a vessel of the size of the radial artery; an opinion strikingly corroborated by the views and doctrines of Dr. Hope, as detailed in a preceding article. Whatever doubts may be entertained of the possibility of such minute discrimination, the general principle, that the pulse should always be considered in two points of view—as an index to the state of the heart namely, as well as that of the system—is, we conceive, of great practical importance.

This chapter contains good general precepts, both for the hygeienic and therapeutical treatment of old age, which might suggest materials for a complete treatise on a subject too little attended to.

There are several chapters on the application of particular classes of medicines—as emetics, opiates, digitalis, and antimonial medicines. We have already given a specimen of our author's manner of treating such subjects in our notice of the chapters on diaphoretics and purgatives. Those above enumerated we are obliged to pass by; but there is yet one chapter on the "Uses of Diluents," on which we must pause for a moment, if it were only to solicit attention to a class of remedies as much neglected by British practitioners as they are rendered unduly prominent by our continental brethren. Dr. Holland views their operation under three principal conditions: First, as mechanically diluting and washing away excrementitious or noxious matters from the alimentary canal; secondly, as modifying certain morbid conditions of the blood; and, thirdly, as affecting various functions of secretion and excretion, especially those of the kidneys and skin. We recommend his remarks on all these heads to the attention of the practitioner, and especially one included under the first, though not strictly referrible to it, namely, the internal use of cold water as a local means of refrigeration. He very truly observes, that the abstraction of heat from an inflamed or irritable internal membrane is often quite as salutary as the direct application of cold to a hot and dry skin, although the former use of the remedial agent is, for obvious reasons, more limited. He adds, "I have seen enough of the benefit from cold liquids freely given in the acute stage of gastric disorders, inflammatory and febrile, with express reference to this point of temperature, to justify the recommendation of more frequent recourse to it in practice." We are happy to find so good authority in support of a practice suggested by common sense, and of the utility of which our own experience has amply convinced us.

Chap. xxii. is on "Morbid Actions of intermittent kind."

As an article in our last Number was occupied with the consideration of intermittent fevers, we may be excused from entering into detail on the subject of this chapter. The observations of the author also are directed chiefly to the theoretical part of it, which, from the absence of sufficient data, is of too vague a character to demand our special attention. The general tendency of Dr. Holland's remarks—which extend to the intermittent disorders of sensation and voluntary motion, as well as to febrile affections of the same type—is to connect, and refer to some common law of periodicity, the various healthy and morbid actions of the system which have a disposition to recur at regular intervals. This is evidently the right track of enquiry; but it is perhaps the most obscure in the whole range of medical science. Not a ray of light crosses it; and the only encouragement to attempt its exploration is the probability that a glimpse of the truth, on any one part of the enquiry, might soon enable us to unveil the whole mystery.

Why is the term of existence so rigidly prescribed for all living beings, and yet so variously for different species? Why are infancy, youth, mature age, and senilitude, so definite in their periods? Why is the term of uterine gestation fixed for every species of animal? Why do the catamenia occur monthly in the human female? Why does an ague or a megrim return at certain intervals? When we can answer any one of these questions unequivocally, we shall most likely have a clue to them all.

It is matter of regret to us that we are unable to afford even the most cursory consideration to a long and very excellent chapter on "the Influence of Weather in Relation to Disease." The author directs his attention to the subject of *weather simply*, as determined by the temperature, hygrometric state, weight, and electrical conditions of the atmosphere, exclusively of chemical change in the air itself, or the admixture of other gases, or of animal or vegetable miasmata. Dr. Holland here evinces much physical knowledge and acute observation; but the subject is vast, and treated only in outline, so that any attempt to condense this chapter would be unprofitable.

Four other chapters on the "Exercise of Respiration," on "Diet and Disorders of the Digestive Organs," on the "present Questions regarding Vaccination," and on "Disturbed Balance of Circulation and Metastasis of Disease," we must also refrain from commenting on. There is another chapter on the "Hypothesis of Insect Life as a cause of Disease," which we must content ourselves with recommending to those of our readers who wish to know all that can be said upon the subject. Dr. Holland states the argument in favour of the insect origin and propagation of pestilential diseases, and especially cholera, as lucidly as could be wished by the strongest advocate of that hypothesis, to which, however, our author by no means professes himself a convert. We must acknowledge that we scarcely consider this hypothesis worthy of grave consideration. If cholera, or similar epidemies, be extended by the migration of insect swarms, these insects must be susceptible of life and activity in all localities, seasons, climates, and temperatures. Now this would be an anomaly so extraordinary, and so opposite to everything hitherto known of the external conditions of animal life, that to adduce such a supposition for the solution of the question as to the spread of epidemies appears to us simply to be attempting to solve what is difficult by what is impossible.

But we must now take leave of our author, which we do with many acknowledgments of the pleasure and instruction we have derived from his work. It is pregnant with information and with thought; and, as such, we heartily recommend it to our readers.

---

